# Topical Lovastatin/Cholesterol as a Potential Field-Directed Therapy for Disseminated Superficial Actinic Porokeratosis: A Case Report

**DOI:** 10.7759/cureus.90507

**Published:** 2025-08-19

**Authors:** Austin J Howe, Thomas J Singer, Thomas E Cramer

**Affiliations:** 1 Dermatology, Lake Erie College of Osteopathic Medicine, Elmira, USA; 2 Dermatology, Advanced Dermatology and Cosmetic Surgery, Fort Myers, USA

**Keywords:** disseminated superficial actinic porokeratosis (dsap), field directed, keratinocytic, mevalonate, mevalonate pathway, porokeratosis, topical, topical cholesterol, topical lovastatin

## Abstract

Disseminated superficial actinic porokeratosis (DSAP) is the most common subtype of porokeratoses, a rare group of disorders characterized by abnormal keratinization. Although the exact etiology is not fully understood, DSAP has been linked to genetic mutations in the mevalonate biosynthesis pathway. Based on this association, topical therapy with a combination of lovastatin and cholesterol has recently emerged as a promising treatment option. In this case study, we present a patient with extensive lower-extremity DSAP and a history of unsuccessful treatment with multiple traditional modalities, who was successfully treated with a 2% lovastatin/cholesterol formulation.

## Introduction

Porokeratoses are a group of skin conditions resulting from disordered keratinization caused by genetic mutations in the mevalonate biosynthesis pathway. These mutations have been reported in both autosomal dominant inheritance patterns and de novo mutations [1]. Several subtypes of porokeratoses have been described, with the most common being disseminated superficial actinic porokeratosis (DSAP). Other well-recognized subtypes include porokeratosis of Mibelli, disseminated superficial porokeratosis, linear porokeratosis, and porokeratosis palmaris plantaris et disseminata [1].

Porokeratosis is recognized as a precancerous condition [2]. The most commonly reported associated malignancy is squamous cell carcinoma (SCC), although cases of basal cell carcinoma and melanoma have also been described [1]. The rate of malignant transformation has been reported to range from 6.4% to 11.6% [2-4].

Clinically, DSAP occurs primarily on sun-exposed areas of the skin and is more common in women than in men. Lesions typically present as well-demarcated annular plaques with a raised hyperkeratotic, desquamating border and a slightly atrophic central area [1]. They may be asymptomatic or pruritic, and symptoms often worsen with sunlight exposure [5]. Porokeratoses are usually diagnosed clinically, although biopsy may be performed as a confirmatory test in atypical or uncertain cases. Histologically, all porokeratoses are characterized by the presence of a cornoid lamella [1].

There is no widely accepted treatment for DSAP, as the rarity of the disorder has limited the performance of large-scale randomized trials. Traditional treatment modalities have included topical and systemic agents such as salicylic acid, topical glucocorticoids, and retinoids, as well as physical therapies such as surgical excision, cryotherapy, and laser ablation [6,7]. However, these conventional approaches are often ineffective, costly, associated with adverse effects, or impractical for patients with DSAP involving large areas of skin [8]. Consequently, DSAP has gained a reputation for being resistant to most standard therapies and particularly difficult to manage.

In recent years, DSAP and other forms of porokeratosis have been linked to mutations in the mevalonate biosynthesis pathway. These mutations are hypothesized to cause cholesterol deficiency within affected tissue, leading to impaired skin barrier function due to insufficiency of the extracellular lipid matrix in the stratum corneum. Additionally, the accumulation of toxic intermediate metabolites may contribute to disease development [8,9]. These findings suggest that cholesterol modulation may represent a promising therapeutic approach.

## Case presentation

A 75-year-old woman presented to the clinic with numerous light brown and erythematous pink macules distributed extensively over the lower extremities and the dorsal surfaces of the feet. On dermatoscopic examination, the lesions appeared annular, with central atrophy and a keratotic ring of desquamation at the periphery.

**Figure 1:**
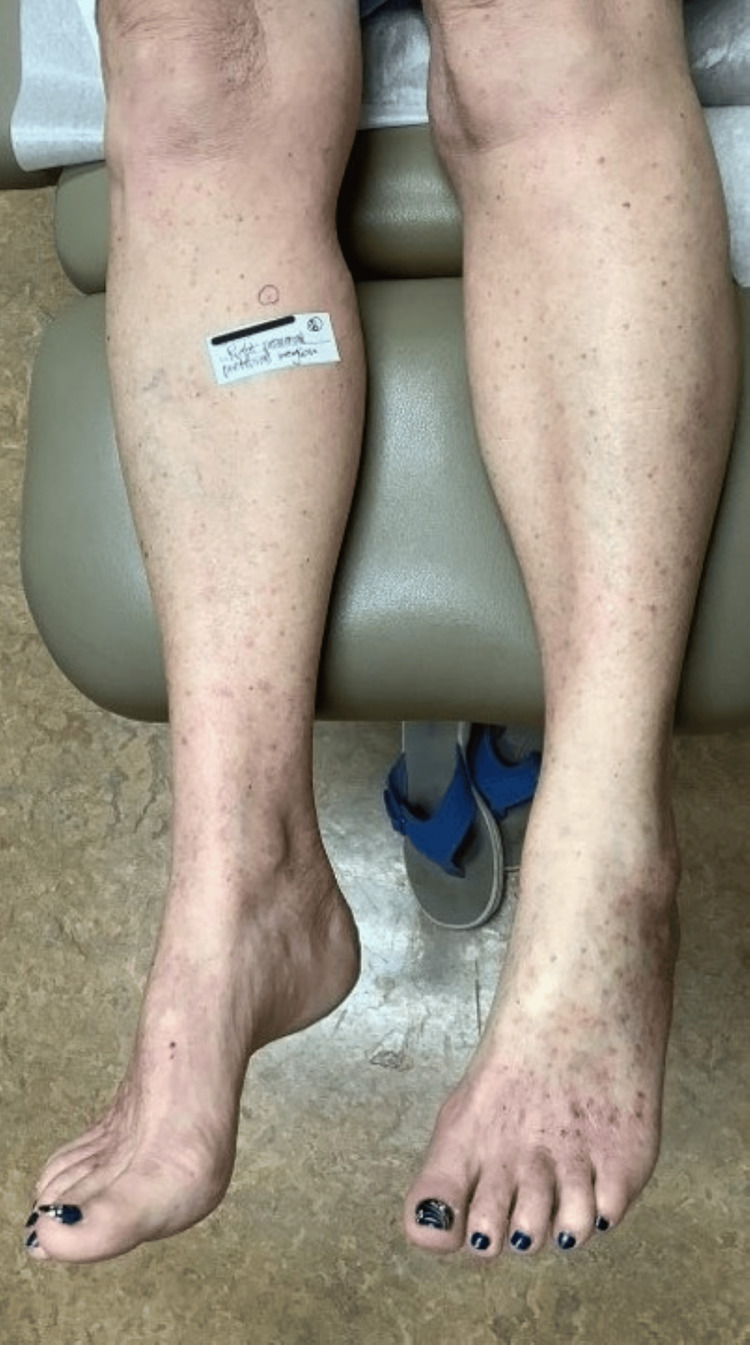
Patient’s legs prior to treatment with lovastatin/cholesterol ointment The circle indicates the site of SCC-IS arising from a porokeratotic lesion. SCC-IS: squamous cell carcinoma in situ

**Figure 2:**
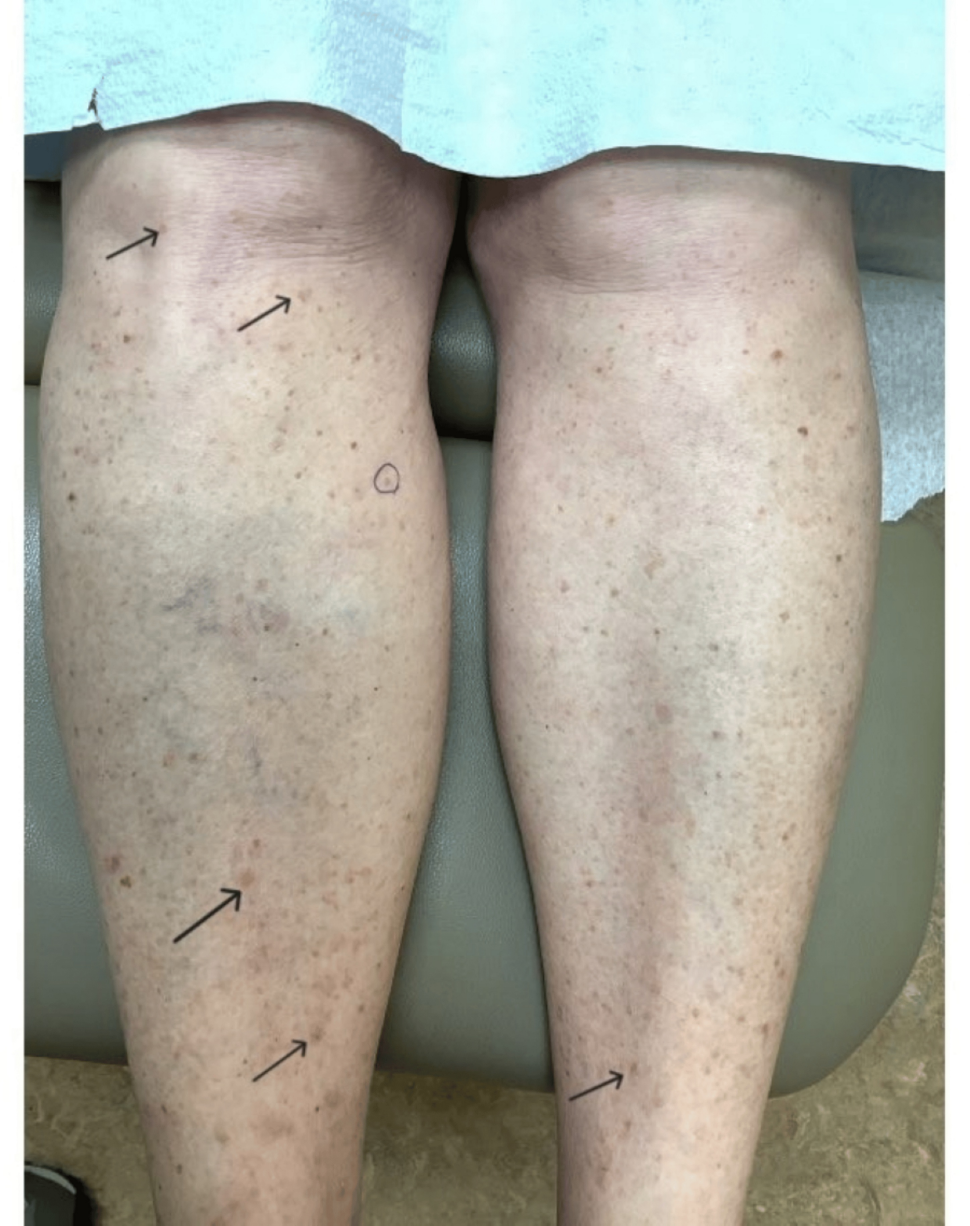
Patient’s shins prior to treatment with lovastatin/cholesterol ointment Black arrows indicate several DSAP lesions. The circle indicates the site of SCC-IS arising from a porokeratotic lesion. DSAP: disseminated superficial actinic porokeratosis; SCC-IS: squamous cell carcinoma in situ

**Figure 3:**
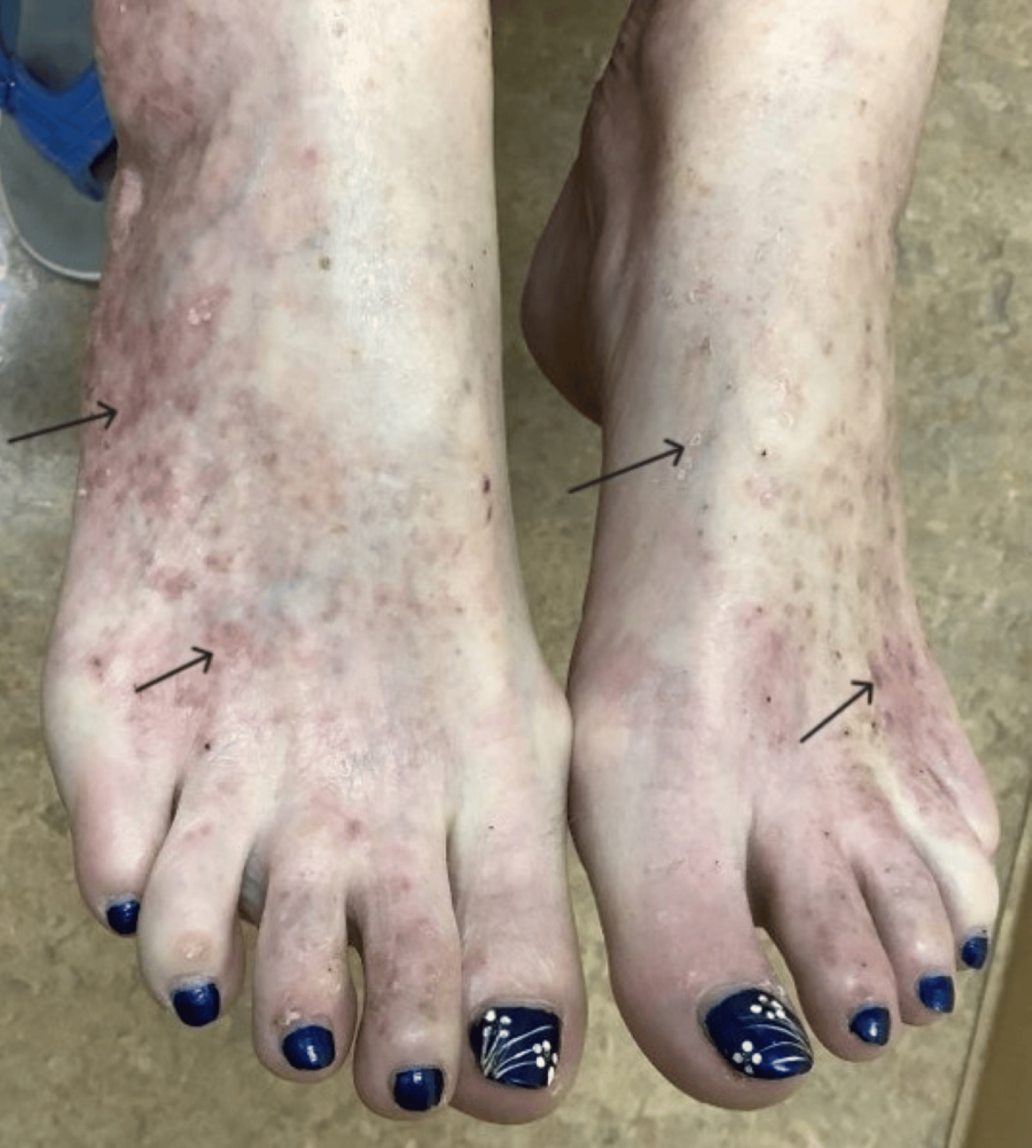
Patient’s feet prior to treatment with lovastatin/cholesterol ointment Black arrows indicate several DSAP lesions. DSAP: disseminated superficial actinic porokeratosis

A clinical diagnosis of DSAP was made based on these findings. Initial treatment with topical 5-fluorouracil did not demonstrate satisfactory improvement (Figure 1, Figure 2, Figure 3). Despite therapy, the patient continued to complain of pruritus and dissatisfaction with the cosmesis of her lesions. Examination revealed a minimal reduction in the number of lesions. Additionally, one lesion demonstrated signs of malignant transformation, and a biopsy confirmed SCC in situ.

Acitretin therapy was then trialed at a dose of 25 mg taken orally every other day, which was reduced to 10 mg after one month. However, this therapy was discontinued three months later after the patient reported muscle pain and hair loss, and laboratory tests showed a mild decline in renal function. Despite this treatment, she continued to experience pruritus, and only an approximately 20% reduction in lesion number was observed. At that point, field-directed therapy was initiated with a pharmacy-compounded topical ointment containing 2% lovastatin/cholesterol, applied twice daily.

**Figure 4:**
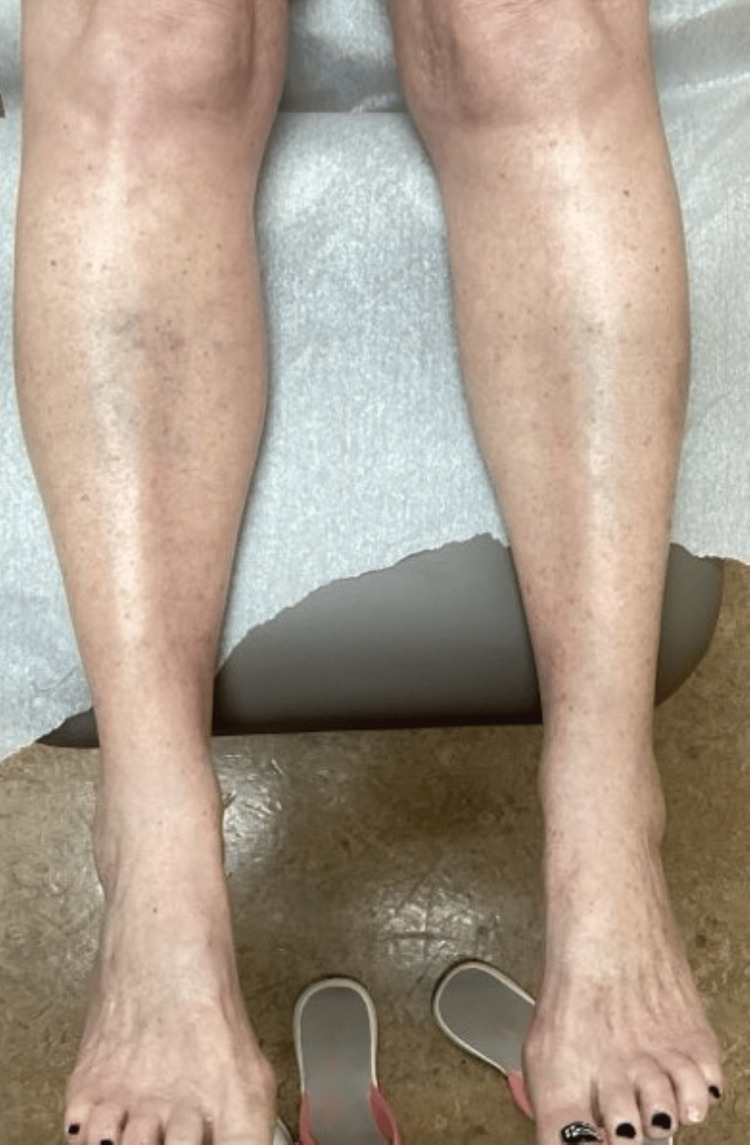
Patient’s legs and dorsum of the feet after one year of treatment with lovastatin/cholesterol ointment

**Figure 5:**
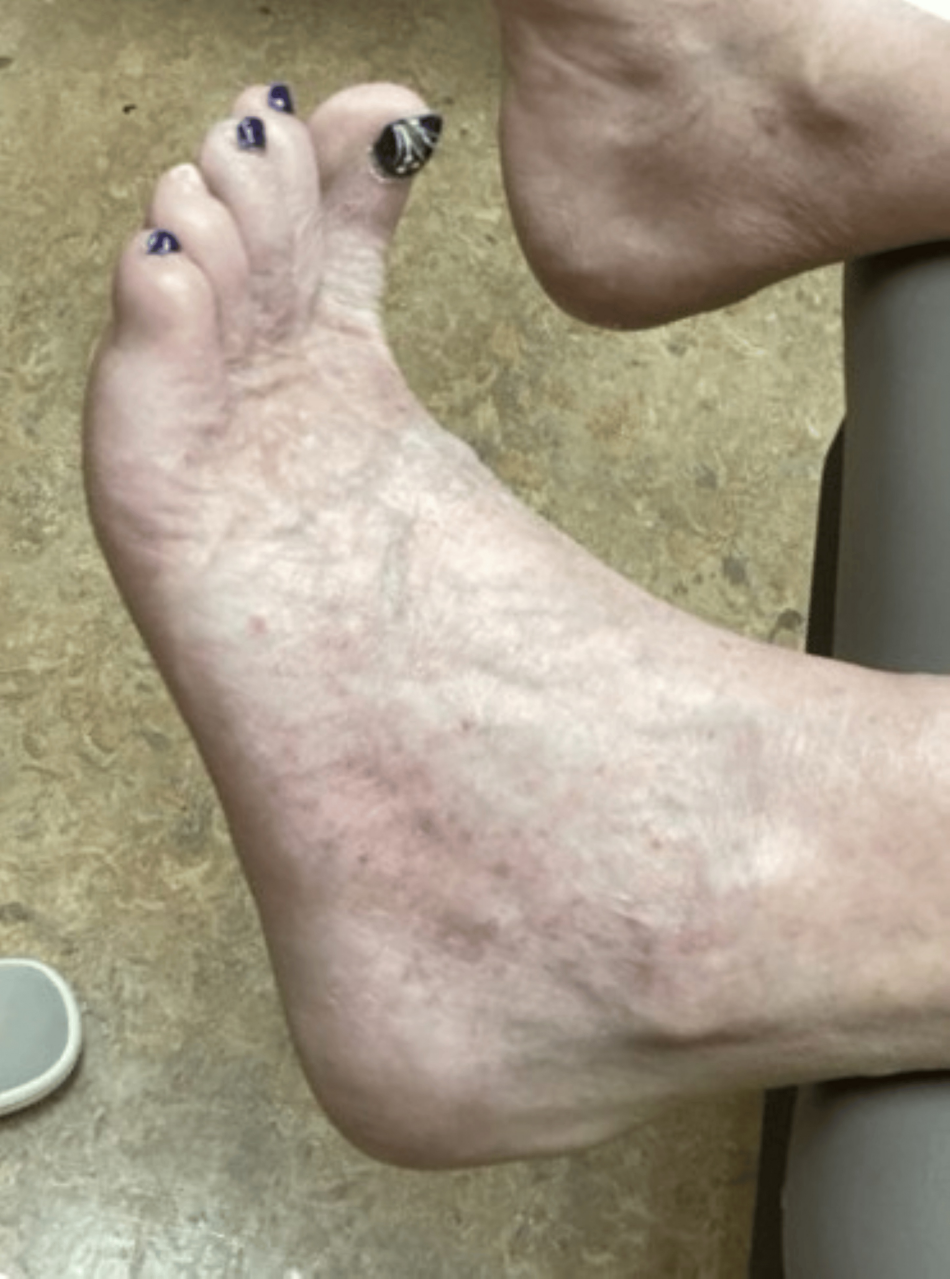
Patient’s left foot after one year of treatment with lovastatin/cholesterol ointment

**Figure 6:**
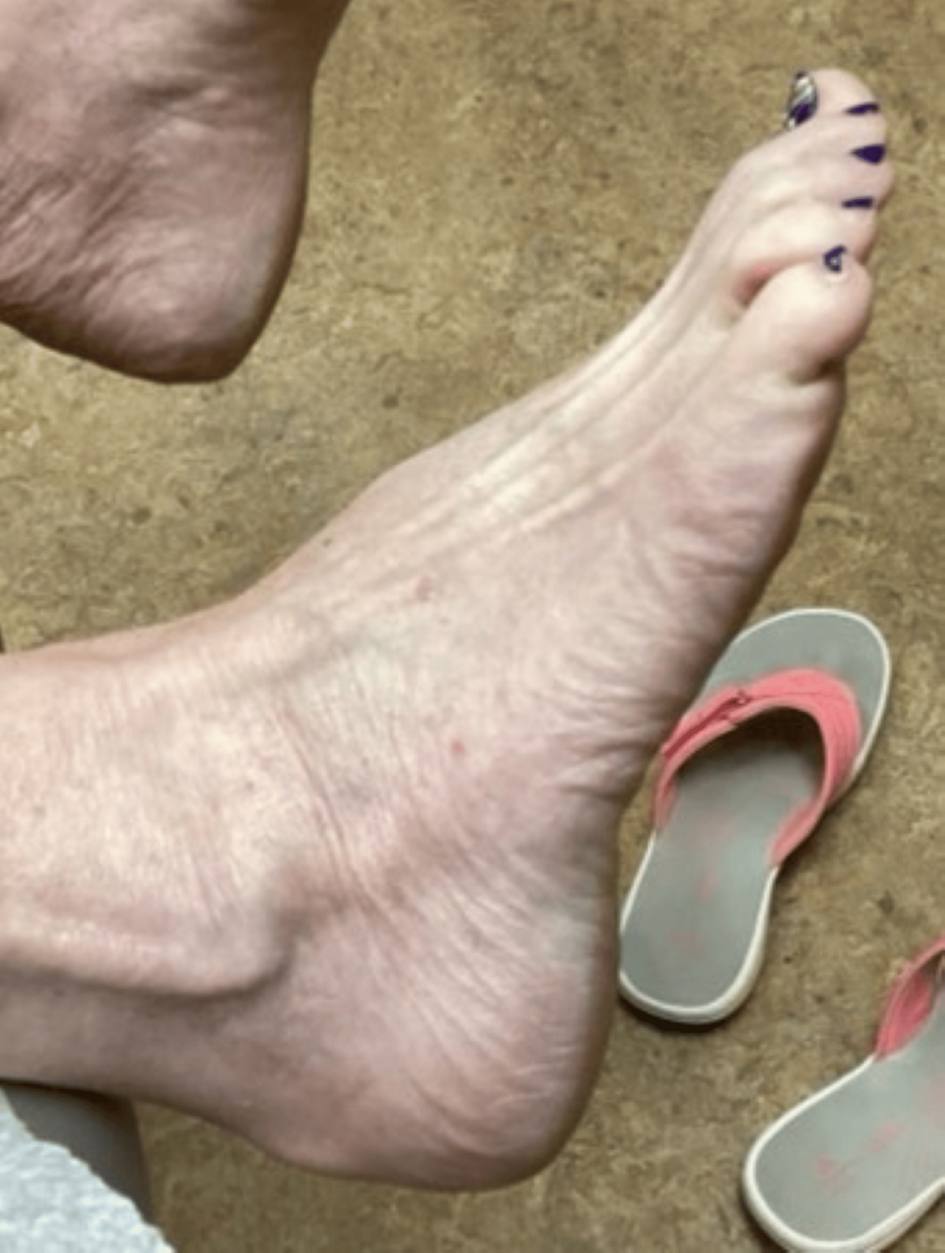
Patient’s right foot after one year of treatment with lovastatin/cholesterol ointment

During follow-up, the patient’s condition had greatly improved. Many of the lesions on her legs had resolved over the first two months of treatment. At the one-year follow-up after initiation of therapy, the patient continued to use the ointment as prescribed and reported no recurrence or flare of lesions during that time. There had been an approximately 90-95% reduction in the total number of lesions since therapy was initiated, and she no longer reported pruritus in her legs and feet (Figure 4, Figure 5, Figure 6). Additionally, no further instances of malignancy were identified in the affected regions, despite regular clinical monitoring with skin examinations every three months. She experienced no adverse reactions to the treatment.

## Discussion

DSAP is a rare, benign pathology that is recognized as a precancerous condition, with a malignant transformation rate ranging from 6.4% to 11.6% [2-4]. The condition is associated with both genetic predisposition and UV damage [5]. Although often asymptomatic, DSAP can cause significant cosmetic concerns and, in cases of widespread distribution, may complicate the identification and demarcation of malignant transformation.

Treatment of DSAP and other forms of porokeratosis has been notoriously difficult due to poor responsiveness, intolerable side effect profiles, and high recurrence rates with traditional therapies [7]. Consequently, recent reports have explored the use of topical statins in combination with cholesterol as a potentially effective alternative that avoids many of the drawbacks of conventional treatments.

This emerging treatment modality is based on the current understanding that porokeratoses result from genetic mutations in the mevalonate biosynthesis pathway, specifically loss-of-function mutations in the mevalonate kinase, phosphomevalonate kinase, and mevalonate diphosphate decarboxylase enzymes. These deficiencies are thought to reduce cholesterol availability in affected tissue, impairing skin barrier synthesis, while also allowing the accumulation of toxic metabolic intermediates [8,9]. In theory, topical statins inhibit HMG-CoA reductase, thereby blocking cholesterol biosynthesis and preventing the production of toxic metabolites, while topical cholesterol supplementation bypasses the defective pathway by replenishing the cholesterol deficit [7,8].

In this case, our patient, who had extensive clinical lesions and biopsy-proven malignant transformation of porokeratotic lesions, had failed multiple traditional treatment modalities commonly used for DSAP. Despite these prior difficulties, she responded remarkably well to this new therapy and has continued treatment for a full year without adverse events, flare-ups, or further malignant transformation.

To date, no large-scale studies have evaluated this emerging treatment for porokeratoses. Several case reports have documented its efficacy, and one randomized clinical trial compared lovastatin/cholesterol with lovastatin alone in 24 patients. Across these studies, improvement in lesions was consistently observed [8,10-13]. In the randomized trial, however, no significant difference was found between the combination therapy and lovastatin alone, suggesting that the statin itself may be the key therapeutic agent and that cholesterol supplementation may be unnecessary [11]. Additionally, a recent case report described allergic contact dermatitis in association with topical simvastatin treatment for porokeratosis [14].

This study is not without limitations. As a single-patient case report, its findings cannot establish efficacy. Furthermore, due to the widespread distribution and large number of lesions, precise monitoring was not feasible, and reductions were estimated using percentages over the treatment course. Additional large-scale studies are needed to better evaluate the efficacy of this therapy. Future research may also explore whether topical statins could be useful in other keratinocytic disorders, given their apparent success in DSAP and favorable safety profile.

## Conclusions

Traditional treatment modalities for DSAP are limited by poor efficacy, side effects, and lack of robust evidence, preventing the establishment of a gold-standard therapy. This case supports the possibility that topical lovastatin/cholesterol may represent a promising alternative for long-term management. Such treatment may reduce the burden for both providers and patients by offering easier administration, a more favorable side effect profile, and lower cost.
